# Sleep-Effects on Implicit and Explicit Memory in Repeated Visual Search

**DOI:** 10.1371/journal.pone.0069953

**Published:** 2013-08-02

**Authors:** Thomas Geyer, Hermann J. Mueller, Leonardo Assumpcao, Steffen Gais

**Affiliations:** 1 Department Psychologie, Ludwig-Maximilians-Universität, München, Germany; 2 Department of Psychological Sciences, Birkbeck College, University of London, London, United Kingdom; University of Bath, United Kingdom

## Abstract

In repeated visual search tasks, facilitation of reaction times (RTs) due to repetition of the spatial arrangement of items occurs independently of RT facilitation due to improvements in general task performance. Whereas the latter represents typical procedural learning, the former is a kind of implicit memory that depends on the medial temporal lobe (MTL) memory system and is impaired in patients with amnesia. A third type of memory that develops during visual search is the observers’ explicit knowledge of repeated displays. Here, we used a visual search task to investigate whether procedural memory, implicit contextual cueing, and explicit knowledge of repeated configurations, which all arise independently from the same set of stimuli, are influenced by sleep. Observers participated in two experimental sessions, separated by either a nap or a controlled rest period. In each of the two sessions, they performed a visual search task in combination with an explicit recognition task. We found that (1) across sessions, MTL-independent procedural learning was more pronounced for the nap than rest group. This confirms earlier findings, albeit from different motor and perceptual tasks, showing that procedural memory can benefit from sleep. (2) Likewise, the sleep group compared with the rest group showed enhanced context-dependent configural learning in the second session. This is a novel finding, indicating that the MTL-dependent, implicit memory underlying contextual cueing is also sleep-dependent. (3) By contrast, sleep and wake groups displayed equivalent improvements in explicit recognition memory in the second session. Overall, the current study shows that sleep affects MTL-dependent as well as MTL-independent memory, but it affects different, albeit simultaneously acquired, forms of MTL-dependent memory differentially.

## Introduction

An important feature of the human brain is its ability to adapt to repeated stimulation by extracting recurring information and modifying behavior accordingly. Nearly all brain systems concerned with perception, cognition, and action control have this adaptive ability in one way or another. Previous research has identified different memory systems, which are distinguishable according to how and what information is stored about previous experiences. Typically, different types of tasks are used to investigate different forms of memory. However, there are paradigms that permit multiple forms of memory to be examined simultaneously. One of these paradigms is ‘contextual cueing’. Contextual cueing refers to the phenomenon that visual search is facilitated by repeated presentation of target-distractor configurations, compared with novel, non-repeated arrangements. That is, search reaction times (RTs) are usually faster for repeated relative to non-repeated displays – an effect which emerges after some 100–150 trials on the task and can persist for several days [1], [2]. This benefit is usually attributed to implicit perceptual memory for spatial (configural) target-distractor contexts, guiding focal attention more rapidly to the target location [3], [4]. Interestingly, contextual cueing has been shown to occur only for a limited number of repeated displays [5]–[8]. Furthermore, when asked to explicitly recognize previously presented target-distractor configurations, observers are able to reliably tell apart at least some of the repeated from non-repeated displays [5], [8]. Importantly, Geyer et al. [5] also showed that contextual cueing can manifest for a given display independently of whether or not this item is explicitly recognized. This suggests that context-dependent configural learning and explicit knowledge of repeated configurations are supported by separate memory processes.

Other investigations of contextual cueing have shown that the effect is highly flexible. Target-distractor contingencies acquired with a particular set of stimulus attributes can transfer to other stimulus attributes. Such transfer effects have been reported for stimulus features, dimensions, modalities, and the specific search task performed by observers [3], [9]–[11]. These findings suggest that contextual cueing is independent of the search items’ perceptual attributes, but rather supported by a spatial long-term memory that stores associations between the target and the distractor arrangement, or between individual target-distractor pairs [12], [13]. Regarding the brain mechanisms underlying contextual cueing, it has been demonstrated that the medial temporal lobe (MTL) supports learning of repeated configurations in visual search (for a review, see [14]). A number of recent fMRI studies provide evidence that the perirhinal and entorhinal cortices in particular are contributing to contextual cueing [15], [16]. Moreover, patients with lesions to the MTL also show impaired contextual cueing [17], [18]. Importantly, the neural substrates of contextual cueing are different from those mediating other aspects of visual search, such as attention or gaze control, with the latter being supported by neocortical (e.g., frontal and parietal eye fields) and subcortical (e.g., superior colliculus and thalamus) brain structures (for a review, see [19]).

Given the bulk of evidence pertaining to the issue of sleep-dependent consolidation in the domains of declarative (e.g. [20]) and procedural memory (e.g. [21]), recent studies have examined whether different forms of memory within these domains are differentially affected by sleep. For instance, it has been suggested that recall is more strongly influenced by sleep than recognition [22], and that awareness modifies the degree to which procedural memory benefits from sleep [23]. Contextual cueing lends itself particularly well to the investigation of such differential aspects of memory for several reasons. First, using this paradigm, procedural learning can be studied by examining practice-dependent gains in mean RTs both within and across experimental sessions. Second, implicit, MTL-dependent contextual cueing can be investigated by comparing RT performance between repeated and non-repeated search displays. Faster RTs are expected for repeated displays, because display repetition promotes the acquisition of an implicit spatial-associative memory for the item configuration. Third, explicit knowledge of repeated configurations learned during a visual search task can be tested with an explicit recognition task [5], [7]. Crucially, these three types of memory reflect independent processes. Contextual cueing and explicit recognition occur for separate subsets of repeated displays, whereas procedural learning can be observed for non-repeated as well as for repeated displays.

In the current study, we examined the role that sleep plays for these three separable forms of memory, tested within a single contextual cueing task. Specifically, the present experiment asked whether a period of sleep, compared with a period of controlled rest, has a positive influence on the number of displays that generate contextual cueing (implicit configural memory), the number of displays that are explicitly learned (explicit recognition memory), and, respectively, the general facilitation of RT performance (implicit procedural memory). Although previous studies have reported effects of sleep on explicit and implicit learning as well as on MTL-dependent and -independent forms of learning, the experimental conditions were often not readily comparable, owing to differences in, for example, the amount and circadian timing of sleep and the level of initial learning. By employing a task that induces different types of learning with the same stimuli, we were able to compare the effects of sleep on different memory systems under exactly the same experimental conditions.

## Method

### Ethics Statement

The experiment was approved by the ethics committee of the Department of Psychological Sciences, Birkbeck College, University of London.

### Participants and Procedure

26 unpracticed observers volunteered to participate in the study (9 women, mean age: M = 28.80, SD = 3.86 years). 15 observers were undergraduate and postgraduate students from Birkbeck College, Queen Mary’s College, and University College London (University of London), who volunteered their services within an informal inter-collegiate participants exchange scheme. The remaining 11 observers were recruited from various backgrounds outside the academic environment and paid at a rate of Euro 10 per hour. Observers were recruited via university advertisement and social networks. All observers had normal or corrected-to-normal vision and had no history of neurological, psychological, or any other chronic illnesses. All observers gave written informed consent prior to their participation. Participants were instructed to have between 7 and 8 hours of sleep the two nights preceding the experiment. Further, they were told not to drink caffeine or alcohol 24 hours prior to and during the experimental day. Before the experiment, they filled in a short questionnaire assessing the quality of their nocturnal sleep preceding the experimental day. They entered their ratings on a 5-point scale, ranging from 5 (very good) to 1 (very bad). Overall, the ratings did not differ between the two experimental groups: nap, M = 3.46, SD = 0.96, versus rest: M = 3.76, SD = 1.01.

The experiment consisted of a training and a test session, both conducted in a quiet and dimly lit laboratory cubicle at Birkbeck College, Department of Psychological Sciences. During both sessions, participants performed a contextual cueing task. The first session took place in the morning between 9 am and 10 am and lasted about 50 minutes; the second session in the evening between 5 pm and 6 pm, lasting 15 minutes. After the first session, participants were randomly assigned to a nap (N = 13) or rest group (N = 13). The use of a between-subject design was motivated by earlier findings from the contextual cueing task, suggesting that the effect is highly affected by proactive interference [24]–[26]. Between 12.30 pm and 2.30 pm, participants in the nap group were required to take a nap of about 80 minutes. The nap was taken at the participants’ homes. During sleep, the experimenter stayed in an adjacent room and noted the time in bed. After sleep, subjects reported their estimated sleep duration and sleep quality via questionnaire. Although the home sleep setting lacks polysomnography, sleeping in the habitual environment generally shows better sleep quality compared with laboratory sleep and an absence of the first-night effect (e.g. [27], [28]). Mean time in bed was 79±8 min (± SEM), mean sleep duration was 63±5.8 min. Nap quality was rated 3.6±0.3 on a scale from 5 (very good) to 1 (very bad). Participants in the rest condition were instructed not to sleep during the day. Between 12.30 pm and 2.30 pm, they returned to the laboratory for an 80-minute period of quiet rest, during which they listened to classical music. The rest period was visually monitored by the experimenter in order to ensure that participants did not fall asleep. Observers were tested individually and received written and verbal task instructions.

### Task

**Figure 1:**
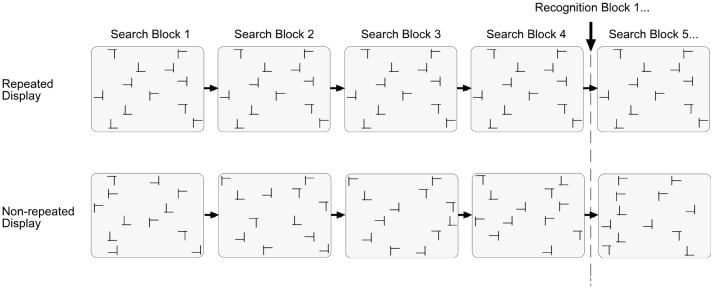
Illustration of the displays used in the present study. Search and recognition trials were presented alternatingly within each session. Half of the trials contained repeated displays (top panel) and the other half non-repeated displays (bottom panel). A recognition test was administered after every fourth (session 1) or every third block (session 2) of search trials. Note that repeated and non-repeated displays were randomly intermixed with each other in a given experimental session.

During experimental sessions, observers completed a contextual cueing task, which involved visual search for a target letter “T” presented amongst distractor letters “L” oriented in various orthogonal directions. On a half of the trials, certain displays were repeatedly presented, i.e., the target and the distractors appeared at identical locations. By contrast, on non-repeated trials, only the location of the target, but not that of the distractors, was repeated across search displays. Thus, the target-distractor configuration was identical across trials only in the repeated condition (while controlling for target location effects in repeated vs. non-repeated displays). Figure 1 shows an example of the search displays. Each experimental trial started with the presentation of a black fixation cross, for 500 ms, in the middle of the monitor (size: 0.72°×0.72° at a viewing distance of approx. 60 cm; luminance: 0.5 cd/m^2^). After a blank interval of 200 ms, the search items appeared. The stimuli consisted of black T’s and L’s (1.22°×1.22°; 0.5 cd/m^2^). Targets were T’s rotated by 90° or 270°, and distractors L’s rotated by 0°, 90°, 180°, or 270° from the vertical (clockwise direction). The L distractors had a relatively large offset (0.27°) at their line junction, increasing their similarity with the target and making search relatively difficult [29]. Each search display consisted of 12 stimuli, which were randomly scattered across the cells of an invisible 8×6 matrix (matrix size: 19.24°×14.43°). There were two restrictions: (1) each of the four quadrants contained three stimuli; and (2) the target appeared equally likely in any of the four quadrants. The placement of the stimuli within the display matrix was slightly jittered, with the horizontal and vertical distances between adjacent stimuli varying randomly between 1.19° and 1.51°. The observer’s task was to find the rotated target letter T and indicate its orientation (left vs. right) by pressing the corresponding key on the computer keyboard (“X” and “N” keys). Observers were instructed to respond as fast and as accurately as possible. Error feedback was provided visually by the presentation of the word “Error”, in black letters, in the screen center. The inter-trial interval was 500 ms and increased to 1000 ms following error trials. Stimuli were presented on a portable PC, with a 17-inch monitor (1920×1080 pixels display resolution), running under the Windows XP operating system. The experimental control software was purpose-written in C++.

At the beginning of the first session, participants practiced the experimental task on a total of 24 non-repeated trials (data not recorded). Then they performed the search task, which consisted of 576 search trials, divided into 24 blocks of 24 trials each. The repeated condition contained 12 randomly arranged target-distractor configurations, generated at the beginning of the search task. These were repeatedly presented on randomly selected trials throughout the search task, with the restriction that each repeated display was shown only once per block. Non-repeated displays were generated online on a given experimental trial. In half of the trials, a repeated arrangement was presented, and a non-repeated arrangement in the other half. To equate target location repetition effects between the two types of displays, the target appeared equally often at each of 24 possible locations throughout the experiment: 12 locations were used for repeated, the other 12 locations for non-repeated displays. After every forth block, observers performed a “yes-no” recognition test, designed to examine whether they could explicitly discern repeated from non-repeated displays. Each of the six recognition tests consisted of 24 trials: 12 “old” (i.e., repeated) and 12 “new” (i.e., non-repeated) displays, presented in randomized order. This yielded a total of 144 recognition trials. On these trials, participants had to indicate whether or not they believed having seen a given display already in the search task, by pressing the corresponding key on the keyboard (“X” key: “Yes, I have seen this display already in the search task”; “N” key: “No I haven’t seen this display in the search task”). Observers were alerted to the respective task (search or recognition) via instruction messages presented in different colors at the start of the relevant block of trials (i.e., “search task” – red color, “recognition task” – green color). Participants were informed neither about the repetition of some displays nor about the insertion of recognition tasks at the beginning of the experiment. The second, evening session consisted of 144 search trials divided into 6 blocks of 24 search trials, and 3 blocks of 24 recognition trials (one after each second block of search trials). Importantly, for a given participant, the same repeated displays were shown in the two experimental sessions.

### Statistical Analysis

The general approach taken in the data analysis was to compare RT and, respectively, recognition performance in the first and second session between the nap and the rest group. Three types of memory were investigated. Implicit contextual cueing was measured by the number of repeated displays that generated a contextual cueing effect. A repeated display was classified as generating contextual cueing if RTs for this display fell below the 99% confidence interval of the observer’s mean RT for non-repeated displays. This conservative 99% criterion was adopted to avoid false positives due to the number of comparisons [5]. In addition, we determined the magnitude of contextual cueing by calculating the difference in RTs between repeated and non-repeated displays. However, mean RTs often vary substantially among observers, which makes it difficult to detect between-group differences. Explicit recognition was measured by means of the sensitivity score d’, i.e., Z_hits_–Z_falsealarms_ [30]. The hit rate was calculated from correctly recalled repeated displays, the false alarm rate from erroneously recognized non-repeated displays. A repeated display with a corresponding d’ larger than 1 was considered to be explicitly recognized (a d’ value of 1 corresponds to 69% of correct responses and is usually considered as moderate detection performance). Single display analyses were conducted separately for the first and the second session. Finally, procedural learning was measured in terms of the improvement in mean RTs across the two experimental sessions.

Statistical analysis was based on mixed-design ANOVAs, with group (nap vs. rest) as between-subject factor, and session (1 vs. 2) in addition to display type (repeated vs. non-repeated) as within-subject factors. Experimental blocks were aggregated into six epochs for session 1 and two epochs for session 2, in order to obtain a reasonable estimate of the contextual cueing effect. When comparing memory performance across sessions, only the last three epochs of session 1 were entered in the analysis, because contextual cueing usually emerges only after a certain number of training trials [11]. RTs outside the range of ±2.5 standard deviations from mean RT were discarded as outliers (3.97% of all trials). Trials on which a response error occurred were also excluded from the analysis (1.25% of all trials). Data analysis was performed using R [31].

## Results

### Contextual Cueing

**Figure 2:**
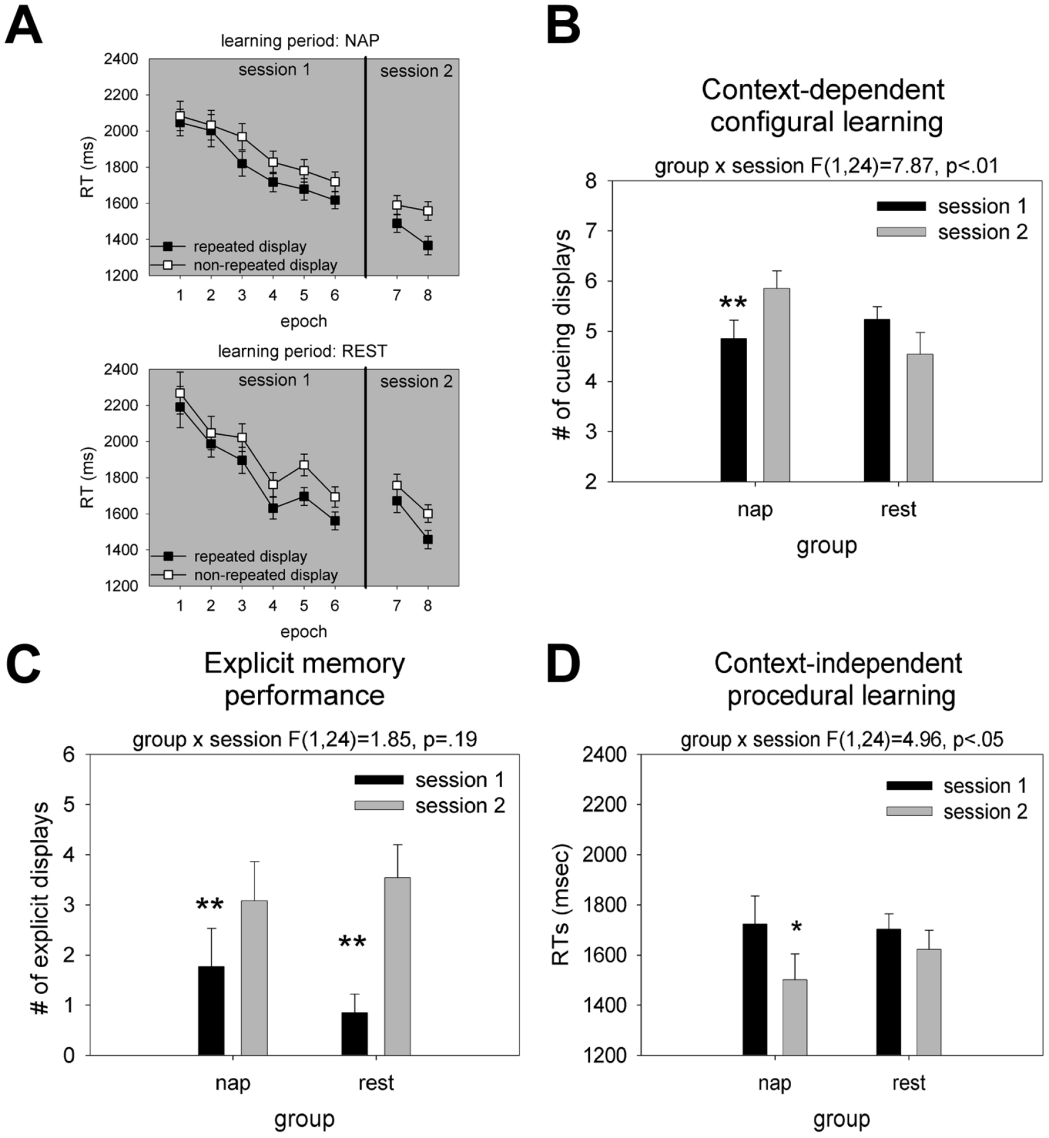
Behavioral performance on the contextual task. **A**: Mean RTs and associated standard errors in the nap (top half) and rest (bottom half) group, for epochs 1–6 (first session) and epochs 7–8 (second session), separately for repeated and non-repeated displays. **B**: Context-dependent configural learning in the nap and rest group. The number of cueing displays is indicated by the black and grey bars, for epochs 4–6 and 7–8, respectively. **C**: Explicit memory performance in the nap and rest group. The number of explicitly remembered displays is indicated by the black and grey bars, for epochs 4–6 and 7–8, respectively. **D**: Context-independent procedural learning in the nap and rest group. RT are indicated by the black and grey bars, for epochs 4–6 and 7–8, respectively. In **B,C** better performance is indicated by higher scores; in **A,D** better performance is represented by lower scores.

**Figure 3:**
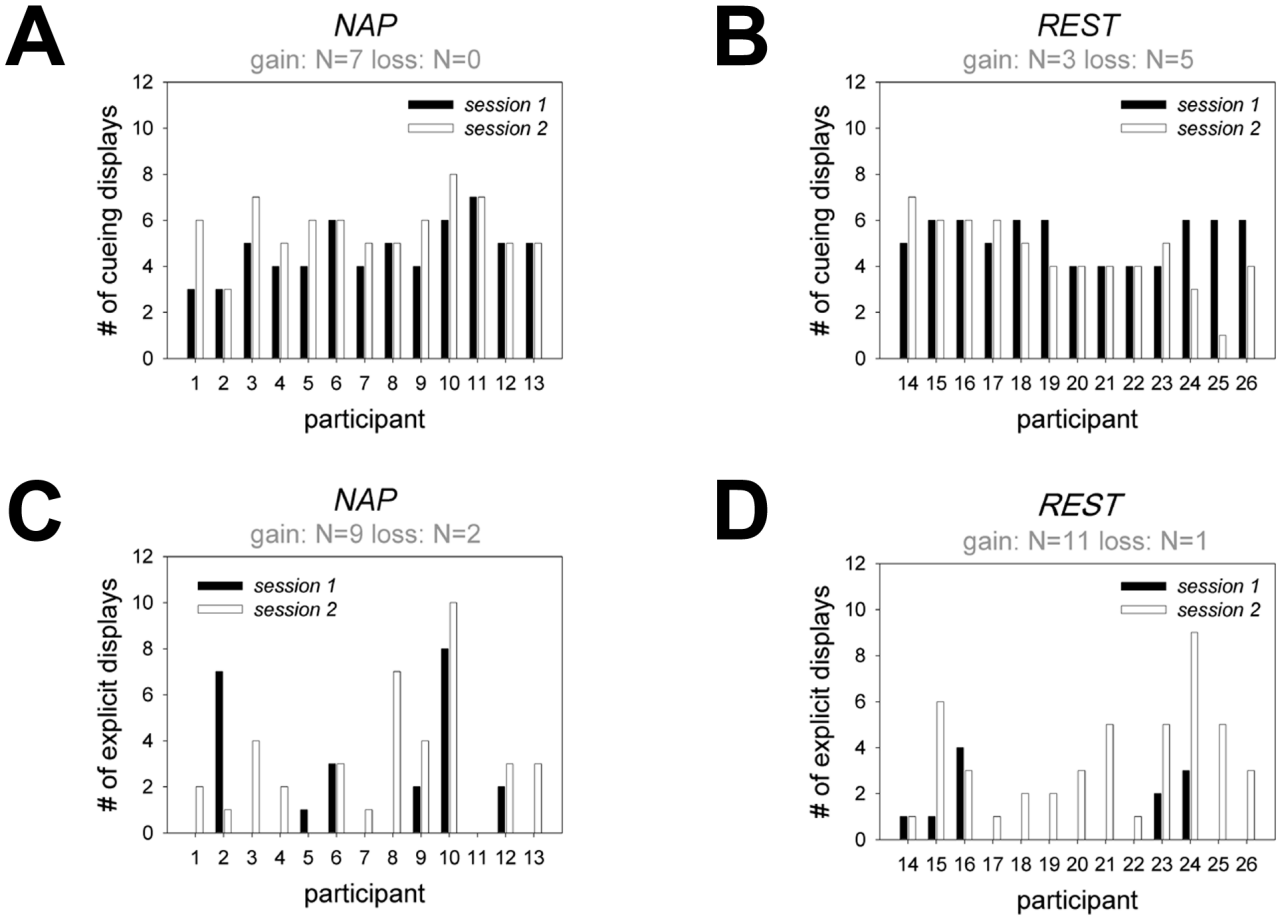
Results from single display analysis. **A**–**B**: Number of repeated displays that generated a contextual cueing effect in the implicit search task for the nap and rest conditions. **C**–**D**: Number of repeated displays that yielded recognition in the explicit memory task. The grey values presented at the top of each graph indicate the number of participants that showed increased (“gain”) or decreased (“loss”) memory performance in the respective learning measure.

Contextual cueing is measured in terms of faster search performance for repeated compared with non-repeated displays. It is indicative of the effect of implicit memory for previously presented target-distractor configurations on search RTs. In the present data, beginning from the third epoch of session 1, a significant contextual cueing effect was observed (see Fig. 2A; all: p<.05; for epochs 1–2: p>.30). We analyzed the number of repeated displays generating contextual cueing in the two groups and sessions. For the sleep group, the number of repeated displays producing a cueing effect increased significantly from 4.86±0.37 (SEM) to 5.85±0.36 (p<.05, see Fig. 2B, 3A). By contrast, in the rest group, the number of cueing displays did not differ between the two sessions. If anything, fewer displays generated a cueing effect in the second session (5.23±0.26 vs. 4.54±0.43, p = .11, see Fig. 2B, 3B). A session×group ANOVA on the number of cueing displays revealed a significant interaction (F_1,24_ = 7.87, p<.01). This interaction was still significant even if the two subjects with the largest performance decreases in the rest group were excluded from analysis (F_1,22_ = 5.39, p<.05). This finding is also illustrated in Figure 3, which shows that distributions of the nap and rest groups are clearly different: whereas 7 out of 13 observers in the sleep group showed an increased number of cueing displays and none showed a decrease, only 3 observers in the rest group showed an increase and 5 showed a decrease.

Overall, in terms of RT, the magnitude of the cueing effect, i.e., RT(non-repeated) - RT(repeated), in the session 1 (last three epochs) was 125±22 ms, and 130±20 ms in session 2. Observers in the nap group showed a numerically larger contextual cueing effect in the second compared with the first session (session 1: 104±38 ms; session 2: 146±16 ms), whereas observers in the rest group showed the opposite pattern (session 1: 145±21 ms; session 2: 114±37 ms). These effects are, however, not significant (both p>.30). A similar finding has recently been reported by Mednick et al. [32], suggesting, at first glance, that sleep does not aid implicit configural learning. However, in view of the large inter-individual variances in RTs, and the significant result of the single display analysis, the failure to find an influence of sleep is likely attributable to the relatively small size of the contextual cueing effect relative to the large variability in RTs across observers. This possibility was examined by an additional analysis. Given a significant correlation between overall response speed and the magnitude of contextual cueing (R = .35, F_1,24_ = 3.83, p<.05), we normalized the contextual cueing effects by dividing them by the individual baseline RTs to non-repeated displays. An ANOVA of these normalized RTs revealed a significant session×group interaction (F_1,24_ = 3.87, p<.05), due to a larger cueing effect in the second than in the first session for the nap group (0.99±0.01 versus 0.43±0.02; p<.05), but not for the rest group (0.67±0.02 vs. 0.80±0.01, p = .69).

In an additional analysis, the correlation between the increase in contextual cueing across sessions (i.e., the difference between session 2 and session 1 in the number of repeated displays generating a contextual cueing effect) and self-reported sleep duration and quality was investigated. A significant correlation was found between change in contextual cueing and sleep duration (R = .53, F_1,11_ = 4.34, p<.05). The correlation between contextual cueing and sleep quality approached significance (R = .48, F_1,11_ = 3.32, p<.10).

### Recognition Memory

Regarding explicit memory, across the two groups, the mean number of explicitly recognized displays was 1.31±0.42 in session 1 and 3.31±0.50 in session 2 (F_1,24_ = 15.45, p<.01). There was no significant session×group interaction (F_1,24_ = 1.85, p = .19, Fig. 2C). In other words, as illustrated in Figure 3C–D, the majority of observers in both groups (nap: n = 9; rest: n = 11) showed improved recognition in the second compared with the first session. Conversely, only a small number of participants showed fewer explicit displays in the second relative to the first session (nap group: n = 2; rest group: n = 1). This pattern suggests that the passing of time as such between the first and second session promotes consolidation of explicit memory for repeated search displays, whether or not participants slept between the two sessions. Thus, in contrast to its function in implicit configural memory, sleep does not improve explicit recognition of repeated search displays in the present task.

#### Procedural memory

Examination of the mean RTs across the six blocks of trials in the first experimental session revealed a steady decrease in RTs from 2,147±129 ms to 1,647±81 ms (F_5,120_ = 27.22, p<.01). This reflects ‘fast’, within session procedural learning of the task, which can occur at various stages of processing, from perception over attention and response selection up to motor response execution. A comparison of the last three epochs of session 1 with the two epochs of session 2 disclosed a pronounced speeding-up of RTs for the sleep group (from 1,722±92 ms to 1,500±99 ms; p<.01), but only a marginal effect for the rest group (from 1,701±70 ms to 1,621±88 ms; p<.07). The session×group interaction was significant (F_1,24_ = 4.96, p<.05, Fig. 2D), confirming the beneficial effects of sleep over wakefulness on procedural learning across sessions.

#### Independence of contextual cueing and explicit recognition memory

The findings of Geyer et al. [5] indicated that implicit contextual cueing and explicit recognition are supported by separate memory processes: repeated displays producing contextual cueing were independent of those that yielded awareness in the explicit recognition task. To corroborate this result in the present data, we analyzed each individual repeated display with regard to whether it did or did not produce contextual cueing and whether it was associated with awareness. Of the repeated displays that generated contextual cueing in the search task, only 8.8±1.81% were also successfully recognized as repeated in the recognition task; likewise, of the repeated displays that did not generate contextual cueing, 10.4±1.85% were successfully recognized in the explicit test. Because the proportion of recognized displays did not differ statistically between displays with and without contextual cueing (8.8% vs. 10.4%; t_25_ = .91, p = .18), both processes can be assumed to be independent.

## Discussion

The present study investigated whether context-dependent implicit learning, context-independent procedural learning, and explicit learning of repeated search displays in a contextual cueing paradigm are equally affected by sleep. These three forms of memory are independent of each other, but are acquired concurrently as a result of repeated exposure to a visual search task. Our results show that implicit memory for repeated displays, which underlies contextual cueing, was significantly greater for the sleep than for the rest group. By contrast, explicit memory for repeated displays improved from test to training session equally across sleep and wakefulness. Finally, context-independent procedural learning was more pronounced when observers had slept before the test session than when they had stayed awake.

Thus, the beneficial effects of sleep in the present contextual cueing task were observed for two independent forms of learning. First, as expected, perceptual learning, as indexed by overall mean RTs to find the target, was larger in the nap than in the rest group. This improvement was independent of a repetition of individual stimuli and thus related to general perceptual and/or motor aspects of the visual search task, such as the coupling of the target onto a response [33]. Similar improvements in procedural memory over sleep have been observed for a number of other tasks, e.g., visual texture discrimination, finger sequence tapping, or mirror tracing. It has been suggested that sleep enhances performance either by actively supporting consolidation of memory traces associated with the task or by removing training-related fatigue [34]–[37]. Second, there was an additional advantage for repeated over non-repeated target-distractor configurations, which was also larger in the nap than rest group. More specifically, for observers having a nap, the number of repeated stimulus displays that generated a contextual cueing effect (i.e., provided effective guidance of attention towards the target location) increased significantly across the two sessions.

It is worth mentioning that both forms of memory are similar in that they require a large number of repetitions during learning and that the resulting memory trace cannot be explicitly accessed. However, both forms of learning differ largely in their underlying neuronal structure. Visual search depends on a large number of different cortical regions, pertaining to perception, attention guidance, working memory, and motor control. Thus, expedited visual search due to procedural learning is likely to be due to more efficient task processing at sites related to visuo-motor and executive processing throughout the brain. Contextual cueing, by contrast, relies on the MTL memory system, in particular the perirhinal and entorhinal cortices [16], [18], [38]. This implies that similar effects of sleep can occur in heterogeneous brain systems. Notably, both tasks were influenced by the same sleep period. MTL-dependent and MTL-independent implicit memory consolidation did not require a different sleep structure in this case.

The third form of memory tested here, explicit learning of individual repeated stimulus displays, was the only process that did not benefit from a nap. That is, explicit memory for repeated displays, which has been shown to depend on parahippocampal and hippocampal structures [16], [39], improved as strongly over a rest interval as over a nap sleep period. Thus, although implicit contextual cueing and explicit recognition are both mediated by MTL structures, these two forms of learning seem to be differentially influenced by sleep. For a number of other explicit, hippocampus-dependent tasks, including verbal and spatial memory, sleep-related improvements have, however, been demonstrated previously [20], [40]. Based on those findings and on animal studies on pattern replay in hippocampal networks (e.g. [41]), the hippocampus was suggested as a core structure for sleep-dependent memory consolidation [42]. On this background, it is surprising that the hippocampus-dependent, explicit memory for repeated displays was the one task that did not benefit from sleep in the present contextual cueing experiments. Given this, we suggest that factors specific to the present experimental design – in particular, a daytime sleep period and a relatively long interval between learning and testing – may have precluded a beneficial effect of sleep on explicit learning. An interaction between sleep and its associated neurohormonal changes, in particular suppression of cortisol, might explain improvement of explicit memory in those nighttime studies. Cortisol is known to influence declarative memory via hippocampal receptors; its reduction during SWS correlates with consolidation during sleep [43]. Daytime sleep, however, does not show this suppression of cortisol release [44]. Thus, for declarative memory, the absence of a benefit from a daytime nap might be related to high cortisol levels. Still, for implicit memory, such an interaction does not seem to be required for sleep effects to occur in the present experiment.

The present experiments were limited with respect to the missing polysomnographic control of sleep. In addition, because subjects were sleeping at home, whereas the rest group was done under controlled laboratory settings, conditions differed slightly between the two groups. However, a number of arguments speak in favor of our observed effects being sleep dependent. First, except for the 80-min period of nap or rest, both conditions were exactly identical. Therefore, either the control visit to the lab impaired performance or the nap enhanced performance. There is no previous evidence that a short rest period under laboratory conditions could impair performance, but many previous studies suggest that sleep could improve performance compared to wakefulness. Second, observed effects cannot be attributed to quiet resting alone, because the control group rested for a period of the same duration as the nap group, but did not sleep. Third, we find positive correlations of sleep duration and quality with the size of the visual cueing effect, which also points to a functional importance of sleep. Differences between nap and control groups must therefore most likely be attributed to sleep.

In summary, a short daytime nap period in between a learning and test session is sufficient to enhance RT performance in a typical procedural visual search task, and an MTL-dependent implicit configural learning task. By contrast, explicit recognition memory, which also depends on the MTL memory system, in particular the hippocampus, does not benefit from a nap. These findings argue for the need to distinguish different forms of memory with regard to whether or not they benefit from sleep, even though they may belong to the same category of perceptual processes (i.e., visual search) or rely on the same brain structure (i.e., MTL cortices).
